# Effect of BaTiO_3_ Filler Modification with Multiwalled Carbon Nanotubes on Electric Properties of Polymer Nanocomposites

**DOI:** 10.3390/nano14141232

**Published:** 2024-07-22

**Authors:** Maxim Sychov, Xingyu Guan, Sergey Mjakin, Lyubov Boridko, Nikolay Khristyuk, Marina Gravit, Semen Diachenko

**Affiliations:** 1 Department of Theoretical Fundamentals of Materials Science, Saint-Petersburg State Institute of Technology, 190013 St. Petersburg, Russia; materials_science_dept@technolog.edu.ru (M.S.); yuihanlyu@yandex.ru (L.B.); nakhristynk@techolog.edu.ru (N.K.); samyon2008@yandex.ru (S.D.); 2 Institute of Silicate Chemistry of NRC “Kurchatov Institute”, 191015 St. Petersburg, Russia; 3 Department of Materials Science, Guiyang University, Guiyang 550005, China; 4 National Research Center “Kurchatov Institute”, Central Research Institute of Structural Materials “Prometey”, 191015 St. Petersburg, Russia; 5 Higher School of Industrial, Civil and Road Engineering, Peter the Great St. Petersburg Polytechnic University, 195251 St. Petersburg, Russia; marina.gravit@mail.ru

**Keywords:** polymer composite, barium titanate, multiwalled carbon nanotubes, dielectric permittivity, lacunarity, scale invariance, percolation

## Abstract

Two ranges of dielectric permittivity (*k*) increase in polymer composites upon the modification of BaTiO_3_ filler with multiwalled carbon nanotubes (MWCNTs) are shown for the first time. The first increase in permittivity is observed at low MWCNT content in the composite (approximately 0.07 vol.%) without a considerable increase in dielectric loss tangent and electrical conductivity. This effect is determined by the intensification of filler–polymer interactions caused by the nanotubes, which introduce Brønsted acidic centers on the modified filler surface and thus promote interactions with the cyanoethyl ester of polyvinyl alcohol (CEPVA) polymer binder. Consequently, the structure of the composites becomes more uniform: the permittivity increase is accompanied by a decrease in the lacunarity (nonuniformity) of the structure and an increase in scale invariance, which characterizes the self-similarity of the composite structure. The permittivity of the composites in the first range follows a modified Lichtenecker equation, including the content of Brønsted acidic centers as a parameter. The second permittivity growth range features a drastic increase in the dielectric loss tangent and conductivity corresponding to the percolation effect with the threshold at 0.3 vol.% of MWCNTs.

## 1. Introduction

The development of polymer-based composites with high dielectric permittivity (*k*) coupled with low dielectric loss is an essential objective for modern electronics [1,2,3,4,5,6,7,8,9,10,11,12], particularly for the fabrication of protective dielectric layers in flexible and stretchable luminescent panels and displays as well as various electric power accumulation devices such as powerful capacitors [13,14,15,16]. Current research [17,18,19,20,21,22,23,24,25,26,27] shows that the desired combination of characteristics of composites may be achieved using barium titanate (BaTiO_3_) as a ferroelectric filler with a high (more than 1000) dielectric permittivity, as well as cyanoethyl ester of polyvinyl alcohol (CEPVA), a mono-component thermoplastic polymer with T_g_~100 °C, as a high-k (*k* ≈ 27) polymer binder [28,29]. Furthermore, as demonstrated in a series of previous studies [28,29,30,31,32,33], in addition to the high permittivity of the components, a necessary condition for achieving enhanced dielectric performances of polymer-based composites is an intensive filler–binder interface interaction resulting in uniform filler distribution in the binder matrix without aggregation of the filler particles. Particularly, in BaTiO_3_-CEPVA composites, the interface activity of barium titanate may be improved through the modification of its surface with various additives, including nanocarbon materials such as graphene, fullerenol, and carbon nanotubes, resulting in the formation of specific centers active towards the sorption of the polymer binder [28,29,30]. These additives were used in previous studies in extremely low concentrations far below the percolation threshold, which is critical because percolation in composites results in a drastic increase in their electrical conductivity and thus deteriorates their dielectric performance. Above the percolation threshold, the dielectric permittivity also abruptly increases according to Equation (1) [34]:

(1) $$k=k_{0}{\left(f-f_{c}\right)}^{-\beta }$$

where *k*_0_ is the permittivity of the matrix, *f* is the concentration (volume fraction) of the electrically conducting component, *f_c_* is the percolation threshold, and *β* is a coefficient depending on the composite structure and interfacial interactions.

Different approaches to control over the filler distribution, aggregation, and percolation phenomena in polymer-based composites involving the introduction of carbon nanotubes were considered in [35,36,37,38]. Particularly, in [35], the addition of magnesium oxide to epoxy resin–MWCNT composites is shown to provide the disaggregation and better dispersion of MWCNTs in the polymer matrix, resulting in a significant increase in electrical conductivity growing with the increase in MgO amount. In [36], percolation in Ni@C (carbon-coated Ni)/epoxy composites is characterized depending on metallic Ni@C particle concentration, the addition of MWCNTs, and the temperature of the material treatment, with the determination of percolation thresholds in the studied composites featuring a drastic increase in both electrical conductivity and dielectric permittivity. In [37], the formation of a microcapacitor network of carbon nanotubes and their compatibilization with a random or block copolymer binder is studied. A similar approach is described in [38], relating to the adjustment of dielectric properties for three-phase nanocomposites involving polyvinylidene fluoride, barium titanate, and carbon nanotubes.

This study presents an investigation of the effect of the surface modification of BaTiO_3_ with carbon nanotubes on the electrical characteristics of CEPVA–BaTiO_3_ composites, using Digital Materials Science methods [39] based on a quantitative analysis of relationships between the numerical (particularly fractal) characteristics of the microstructure of the composites and their target properties. In particular, various factors affecting dielectric permittivity are taken into account and quantitatively characterized, including the content of specific centers on the filler surface, filler–polymer interfacial interactions, and the structure of the composites.

## 2. Materials and Methods

BaTiO_3_ (Fuji Titanium Industry Co., Ltd., Osaka, Japan; particle size approximately 0.5 µm) was modified through the deposition of multiwalled carbon nanotubes (MWCNTs) (MUNT-2; Boreskov Institute of Catalysis of the Siberian Branch of the Russian Academy of Sciences, Novosibirsk, Russia; average diameter 11–12 nm, average length appr. 5 µm, specific surface area 260 ± 5 m^2^/g, *k*~2–4) as an additive capable of the formation of the needed surface active centers. Thus, each MWCNT is able to “attach” several BaTiO_3_ particles and influence the composite’s structure. In order to obtain the composites, the MWCNTs were mixed with barium titanate in different ratios from 0.02 to 0.42 mg MWCNT per 1 g BaTiO_3_. Then, 30 mL of distilled water was added and the mixture was stirred continuously and boiled up to the complete evaporation of water, followed by drying to constant weight.

The surface properties of the initial and modified BaTiO_3_ particles were characterized using the following method. The adsorption of acid–base indicators with different pK_a_ values was accomplished according to a procedure described in detail in previous studies [40,41,42]. The experiment involved spectrophotometric measurements of optical density (*D*) for the following solutions of each indicator at the wavelengths corresponding to their intrinsic absorption maximums:

1. Blank solution, obtained by diluting the initial indicator with distilled water to 5 mL (*D*_0_).

2. Solution prepared according to the same procedure after holding a sample of a weight m_1_ ≈ 20 mg of the analyzed material for 1 h (to establish the adsorption–desorption equilibrium). This measurement gives a changed optical density value *D*_1_ compared to to *D*_0_ due to both the indicator adsorption on the sample surface and pH change after the sample–water contact (caused by the interaction of water molecules with the film surface).

3. A similar sample of a weight m_2_ ≈ m_1_ ≈ 20 mg of the analyzed material placed into a test tube with 3 mL of distilled water for 1 h to establish the equilibrium between water and the sample surface. Then, water was decanted to another test tube, followed by the addition of the indicator (volume *V_ind_*) and distilled water to a total volume of 5 mL. The optical density of this probe *D*_2_ differed from *D*_0_ only due to pH change after sample–water contact, which allowed us to eliminate the contribution of this factor in the differential analysis of the data and consider only the indicator adsorption to determine the content of surface centers with the corresponding pK_a_ value according to the following equation:

(2) $$Q\left(pKa\right)=\left|\frac{\left|D_{0}-D_{1}\right|}{m_{1}}\pm \frac{\left|D_{0}-D_{2}\right|}{m_{2}}\right|C_{ind}V_{ind}∕D_{0}$$

where *C_ind_* is the concentration of the indicator in the solution; *V_ind_* is the volume of the indicator solution taken for analysis; m_1_ and m_2_ are weights of the samples in series 1 and 2, respectively; the «+» sign corresponds to the case where D_1_ and D_2_ are oppositely changed compared to *D*_0_, (*D*_1_ < *D*_0_ and *D*_2_ > *D*_0_, i.e., the changes in optical density caused by adsorption and water–surface interaction are opposite and the decrease in optical density due to the indicator adsorption is greater than the increase due to the water–surface interaction); and the «–» sign corresponds to the one-sided optical density change caused by both adsorption and water–surface interaction (*D*_1_ < *D*_0_ and *D*_2_ < *D*_0_, or *D*_1_ > *D*_0_ and *D*_2_ > *D*_0_, i.e., either the water–surface interaction results in a decrease in the optical density or it results in the growth of this value but the decrease caused by adsorption is not sufficient to compensate for it). In this study, the contents of adsorption centers with pK_a_ values −0.3, 3.5, 4.1, 5.0, and 12.8 were determined using the adsorption of o-nitroaniline, methyl orange, bromo-phenol blue, methyl red, and indigo-carmine indicators, respectively.

Polar ($\delta_{x}^{p}$), dispersive ($\delta_{x}^{d}$), and total ($\delta_{g}^{\Sigma }$) surface energies of composites were determined by measuring the contact wetting angles for water and glycerol according to the following equations:

(3) $$\frac{\delta_{w}^{\sum }(\cos\theta_{w}+1)}{2}=\sqrt{\delta_{w}^{p}}\sqrt{\delta_{x}^{p}}+\sqrt{\delta_{w}^{d}}\sqrt{\delta_{x}^{d}}$$

(4) $$\frac{\delta_{g}^{\sum }(\cos\theta_{g}+1)}{2}=\sqrt{\delta_{g}^{p}}\sqrt{\delta_{x}^{p}}+\sqrt{\delta_{g}^{d}}\sqrt{\delta_{x}^{d}}$$

where *θ_w_* and *θ_g_* are the contact wetting angles for water and glycerol, respectively; $\delta_{w}^{\sum }$ = 72.8 mJ/m^2^, $\delta_{w}^{p}$ = 47.8 mJ/m^2^, and $\delta_{w}^{d}$ = 25.0 mJ/m^2^ are the total, polar, and dispersion surface tensions of water; and $\delta_{g}^{\Sigma }$ = 59.4 mJ/m^2^, $\delta_{g}^{p}$ = 22.4 mJ/m^2^, and $\delta_{g}^{d}$ = 37.0 mJ/m^2^ are the total, polar, and dispersion surface tensions of glycerol.

Barium titanate powder modified with MWCNTs was introduced into the CEPVA polymer solution (PB-paste, Shanghai Keyan Phosphor Technology Co., Ltd., Shanghai, China, *k*~27) at the amount of 1 g modified BaTiO_3_ per 1.1 mL CEPVA solution, and stirred, followed by the agitation of the obtained suspension in a Sapphire ultrasonic bath (PKF Sapphire, Moscow, Russia) for 20 min. The prepared CEPVA/(BaTiO_3_ + MWCNT) suspensions were cast onto 3 × 10 cm glass plates wrapped in aluminum foil and dried at 80 °C for 4 h. Taking into account BaTiO_3_, MWCNT, and CEPVA densities (6.0 g/cm^3^, 2.0 g/cm^3^, and 1.0 g/cm^3^, respectively), the BaTiO_3_ content in all resulting CEPVA/(BaTiO_3_ + MWCNT) composites was calculated as approximately 34 vol.% and kept constant. Then, electrodes of conducting silver-containing paste Contactol were deposited onto the composite layer surface to perform the electrical measurements.

The electrical characteristics of the prepared composites were measured using a E7-20 immittance meter (MNIPI, Minsk, Belarus). The dielectric loss tangent, electrical capacitance, and electrical resistance were measured directly, while the dielectric permittivity *k* was calculated as follows:

(5) $$k=\frac{Cd}{\varepsilon_{0}S}$$

where *C* (F) is the measured electrical capacitance, *d* (m) is the composite layer thickness, *ε*_0_ = 8.854 × 10^−12^ F/m is the dielectric constant of vacuum, and *S* (m^2^) is the surface area of the electrodes.

The structure of the composites was characterized via scanning electron microscopy (SEM) at the Engineering Center of the Saint Petersburg State Institute of Technology, using a Vega 3 scanning electron microscope (TESCAN IC Lab, Brno, Czech Republic).

SEM images were processed using a box-counting method based on the division of images into square cells, followed by the calculation of the number of the filler particles in the cells with a consecutive variation of the cell size [28] to determine the fractal dimension from the slope of the lnN vs. lnx plot, where N is the average number of filler particle mass centers in square cells of size x(μm) in corresponding divisions of the SEM images as shown below; 

Furthermore, the structure of the obtained materials was studied by means of transmission electron microscopy (TEM), using a Tecnai G^2^ F20 S-TWIN + AZtec X-Max 80T electron microscope (FEI Europe B.V., Eindhoven, The Netherlands), and Raman spectroscopy, using a Horiba LabRAM Aramis laser Raman microscope (HORIBA FRANCE SAS, Palaiseau, France).

The impedance characteristics *Z* and the phase angle *θ* were measured using a E7-20 immittance meter (MNIPI, Minsk, Belarus), followed by the calculation of the real (*Z*′) and imaginary (*Z*″) parts of the impedance:

(6) $$Re\left(Z\right)\equiv Z\prime =\left|Z\right|cos\;\theta \;and\;Im\;Z\equiv Z\prime \prime =\left|Z\right|sin\;\theta$$

## 3. Results

### 3.1. Characterization of Dielectric Properties

**Figure 1:**
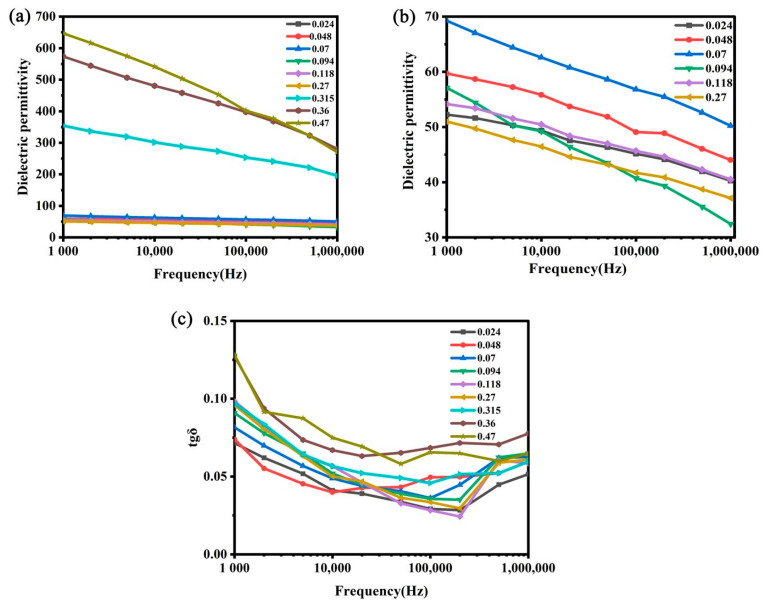
Frequency dependencies of dielectric permittivity for composites with multiwalled carbon nanotube (MWCNT) contents 0.024–0.47 vol.% (**a**) and 0.024–0.27 vol.% (**b**), as well as dielectric loss tangent for composites with MWCNT contents 0.024–0.47 vol.% (**c**).

The frequency dependencies of the dielectric permittivity (*k*) and dielectric loss tangent (tg*δ*) for composites containing BaTiO_3_ modified with different quantities of MWCNTs are shown in Figure 1.

**Figure 2:**
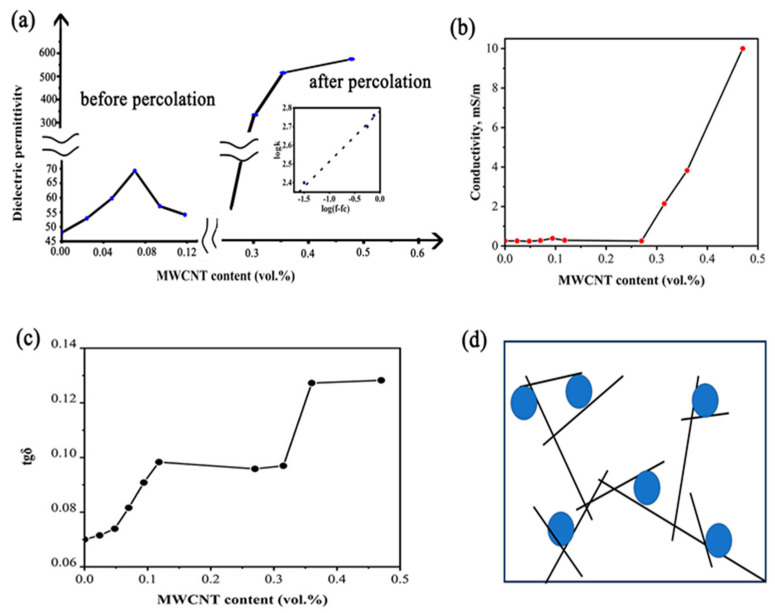
Dielectric permittivity (**a**), specific electrical conductivity (**b**), and dielectric loss tangent (**c**) of the composites as functions of MWCNT content, and scheme of percolation involving MWCNT network (**d**).

From Figure 1, one can see that the change in the electrical properties of composites with the introduction of MWCNTs is not monotonous. Subsequently, for a comparative analysis of dielectric characteristics for composite samples with different MWCNT concentrations, their electrical properties were considered at a frequency of 1 kHz, commonly used for standard tests (Figure 2).

The obtained data indicate the presence of two ranges of permittivity increase depending on MWCNT content:

(1) An increase in dielectric permittivity by approximately 45% (compared to 48 for the MWCNT-free sample) up to a maximum *k* value of approximately 70 at 0.07 vol.% MWCNT content is observed (left insert in Figure 2a), with relatively small increments in dielectric loss and electrical conductivity. As described below, this increase in k value is determined by the improved structural uniformity of the composites due to specific interfacial interactions.

(2) An abrupt increase in permittivity (Figure 2a) coupled with a drastic increase in electrical conductivity at MWCNT contents greater than 0.25 vol.% is observed (Figure 2b), corresponding to percolation in the composite with a percolation threshold f_c_ of approximately 0.30 vol.% of MWCNTs and *β* = 0.3 (Equation (1) and insert in Figure 2a). In Equation (1), *k*_0_ represents the dielectric permittivity of the composite without MWCNTs. The considered percolation is determined by the formation of interpenetrating networks of electrically conductive MWCNTs connected with BaTiO_3_ particles (Figure 2d).

The resulting *β* value is significantly lower compared with reference data on similar three-phase systems and CNT–polymer composites, e.g., *β* = 0.53 for hot-pressed composites of poly(vinylidene fluoride) and MWCNTs added to BaTiO_3_ (20 vol.%) with a particle size of 0.5–1.0 µm [43]. A relatively low *β* value is determined by the effect of BaTiO_3_ particles, occupying a significant fraction of the volume. Therefore, when the concentration of CNTs increases, they can build conducting pathways only in the space between BaTiO_3_ particles, so nanotubes occupy quasi two-dimensional (2D) space between BaTiO_3_ particles, which results in a slower permittivity increase. This effect grows with the increase in BaTiO_3_ content, i.e., the *β* value decreases from more than 1 for CNT–polymer systems [43] with a spatial (3D) CNT structure to approximately 0.53 for 20 vol.% BaTiO_3_ in [44] and approximately 0.3 for 34 vol.% BaTiO_3_ in this research.

(3) Two prominent steps in the dielectric loss tangent growth from 0 vol.% to 0.12 vol.% and from 0.27 vol.% to 0.36 vol.% of MWCNTs (Figure 1c) were observed, indicating two different kinds of structural changes in the composites with carbon nanotubes similar to those described in [42,43,45].

### 3.2. Study of the Filler and Composite Surface Characteristics Correlating with Dielectric Properties of the Composites

**Figure 3:**
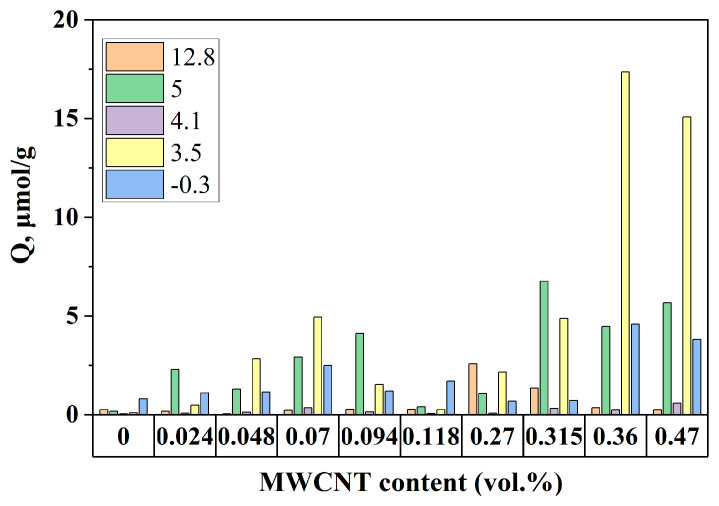
Concentration (Q, μmol/g) of adsorption centers with different pK_a_ values (−0.3, 3.5, 4.1, 5, and 12.8) on the surface of modified barium titanate samples depending on MWCNT content (0–0.47 vol.%).

The concentrations of different adsorption centers on the surface of modified BaTiO_3_ as a function of MWCNT content are summarized in Figure 3.

**Figure 4:**
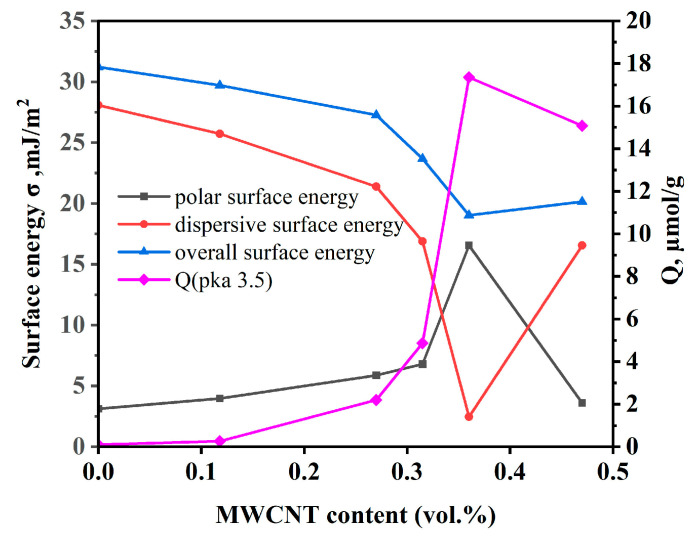
Surface energy (σ, mJ/m^2^) of the composites and content (Q, µmol/g) of Brønsted acidic centers as a function of MWCNT content.

The most significant changes are observed for the Brønsted acidic centers with pK_a_ values −0.3–5.0 corresponding to acidic hydroxyls on the surface of BaTiO_3_. The change in the content of these centers is shown in Figure 4 in comparison with the surface energy of the composites.

**Figure 5:**
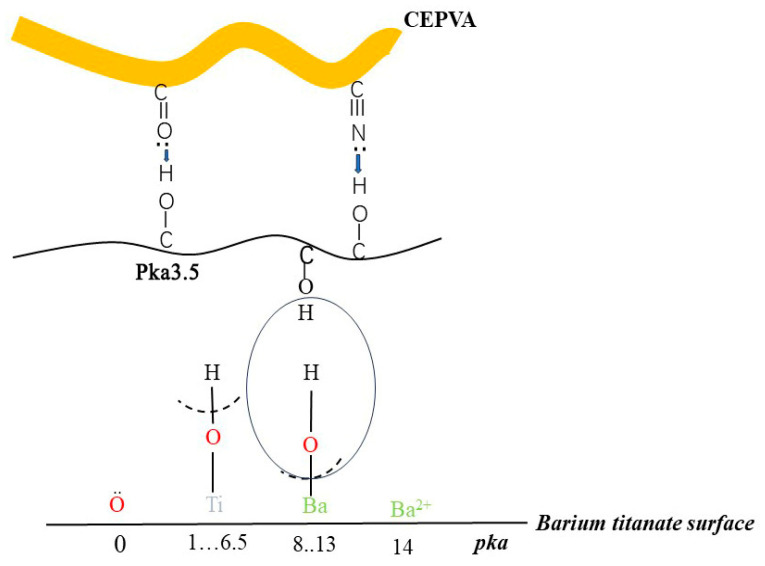
MWCNT-CEPVA-BaTiO_3_ interactions involving surface functional groups.

These data show that the observed increase in the content of Brønsted acidic centers on the surface of BaTiO_3_ is accompanied by a growth of the polar surface energy component of composites up to the maximum at 0.36 vol.%, with the correlation coefficient R^2^ = 0.97. The observed drastic increase in the content of Brønsted acidic centers from almost zero for the initial BaTiO_3_ to about 17 µmol/g suggests that these centers relate to the MWCNT additive. Previously [46], it was shown that Brønsted acidic centers on the surfaces of solids are hydrophilic, which explains the observed increase in surface polarity. The introduction of MWCNTs led to a significant increase in the content of Brønsted acidic centers on the surface of modified BaTiO_3_, providing binding with Lewis basic carbonyl and nitrile groups on the surface of CEPVA, thus promoting filler–polymer interface interactions (Figure 5).

**Figure 6:**
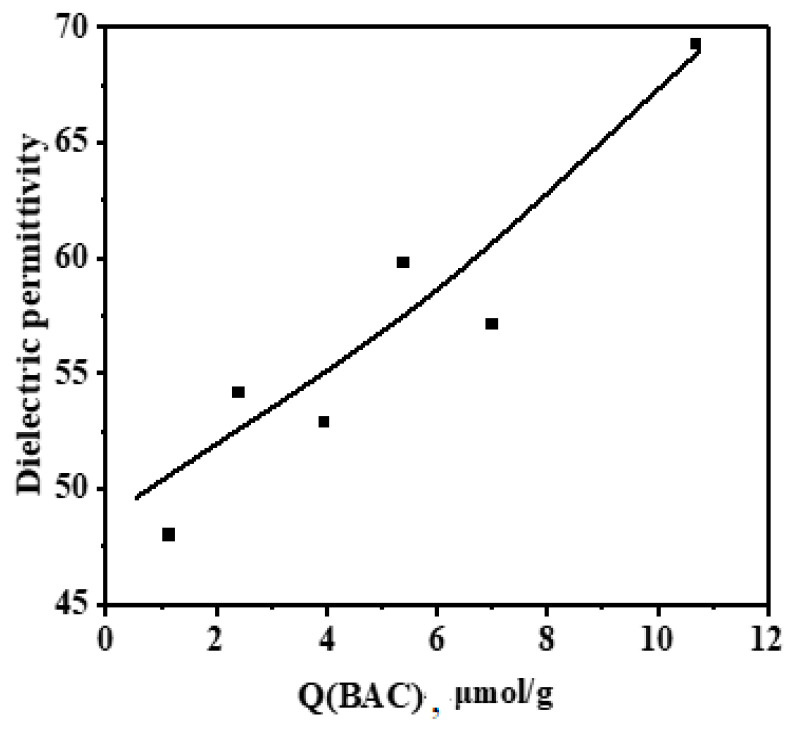
Dielectric permittivity of composites as a function of the content of Brønsted acidic centers (Q(BAC)) on the filler surface (dots) and approximation of these data using a Lichtenecker–Sychov equation (solid line).

These interactions provide a more uniform filler distribution in the matrix, as it will be shown further, thus accounting for the observed correlation between the content of these centers and the permittivity value (Figure 6). Furthermore, the effect of the content of Brønsted acidic centers on the surface of modified barium titanate (Q μmol/g) upon the composite permittivity in the pre-percolation region can be described by a modified Lichtenecker equation, initially expressed as [29]:

(7) $$k^{m}=f_{1}{k_{1}}^{m}+f_{2}{k_{2}}^{m}$$

where *k*_1_ and *k*_2_ are the dielectric permittivity values of BaTiO_3_ (ε ≈ 4400) and CEPVA (*k* ≈ 27), respectively; *f*_1_ and *f*_2_ are the volume fractions of BaTiO_3_ (0.34 vol.%) and CEPVA (0.66 vol.%); and *m* is a coefficient taking into account the composite structure. This equation can be modified [29] by considering m as a linear function of the content of Brønsted acidic centers Q(BAC) on the surface of modified barium titanate that are responsible for the polymer–filler interfacial interactions:

(8) m=a+b∗Q(BAC)

where *a* and *b* are coefficients derived by linear approximations. Thus, the permittivity of the composites is described by the following Lichtenecker–Sychov equation:

(9) $$k^{a+b*Q(BAC)}=f_{1}{k_{1}}^{a+b*Q(BAC)}+f_{2}{k_{2}}^{a+b*Q(BAC)}$$

The approximation of experimental data according to Equation (9) provided a high correlation coefficient of 0.9 (Figure 6).

Thus, the observed correlation between the Brønsted acidic centers content on the modified filler surface and the dielectric permittivity of the composite is determined by the involvement of the corresponding functional groups in the interfacial interactions, improving the filler distribution uniformity in the matrix; see the next section.

### 3.3. SEM Characterization of the Composites and Analysis of Fractal Characteristics

**Figure 7:**
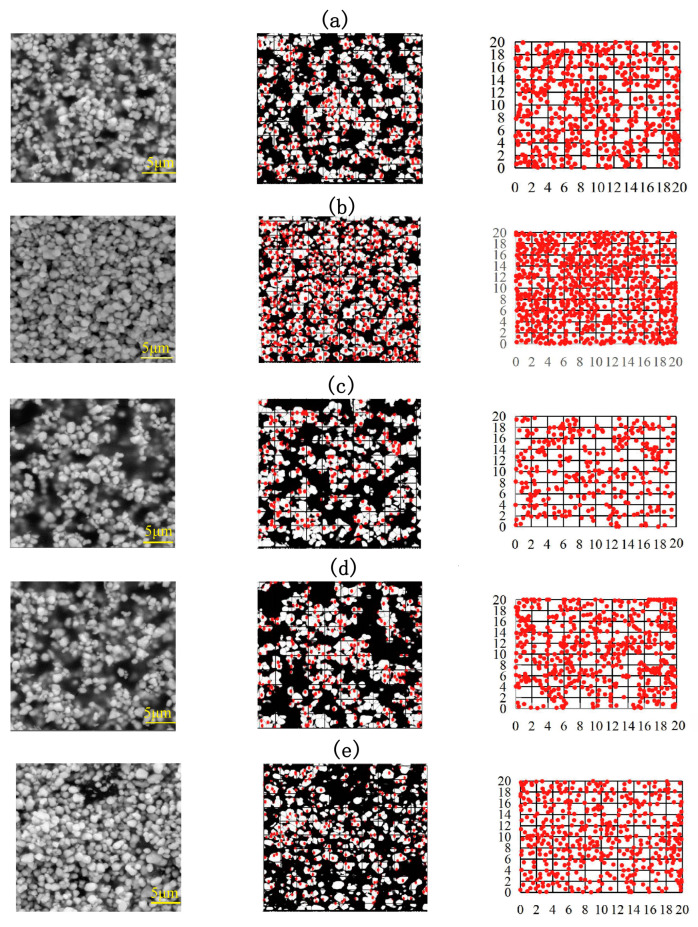
SEM images (**left**), their binarized representations with marked particle centers of mass (**middle**), and distributions of the particle centers of mass in 20 × 20 μm square cells (**right**) for composites with MWCNT contents of 0 (**a**), 0.07 (**b**), 0.12 (**c**), 0.27 (**d**), and 0.36 vol.% (**e**).

To analyze the changes in composite structure caused by filler modification, the composite samples with MWCNT contents of 0, 0.07, 0.118, 0.27, and 0.36 vol.% were characterized via SEM, followed by the processing of the obtained images to derive their fractal characteristics. SEM images of composites, their binarization with marked centers of mass, and exemplary division into square cells are shown in Figure 7a–e. The addition of 0.07 vol.% MWCNTs results in a considerable increase in composite uniformity and filler particle packing density compared with the MWCNT-free composite and samples with higher MWCNT contents. The sample with 0.27 vol.% MWCNTs, corresponding to the near-percolation state, features the lowest packing density; however, when the MWCNT content exceeds the percolation threshold, there is a considerable increase in packing density, resulting in the formation of percolation clusters.

**Figure 8:**
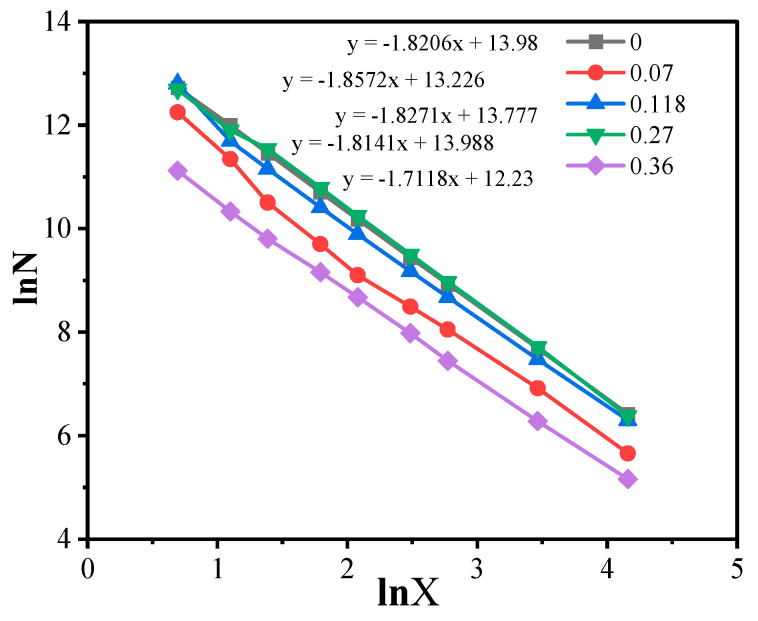
Logarithmic plot of the average number of filler particle mass centers in square cells as a function of their size x for samples with different MWCNT contents (0–0.36 vol.%).

The fractal dimensions of the studied samples were determined as described in the Materials and Methods section from lnN vs. lnx plots exemplarily shown in Figure 8.

**Figure 9:**
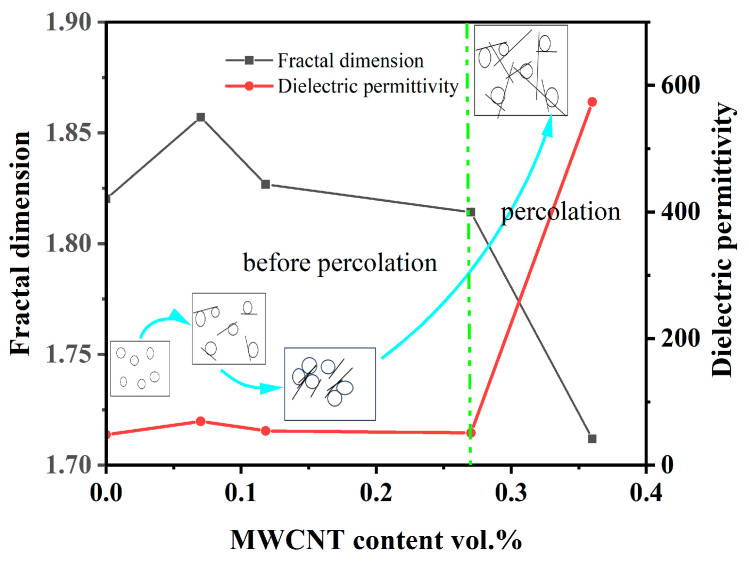
Fractal dimension and dielectric permittivity as a function of MWCNT content.

As one can see from Figure 9, up to the percolation region, there is a good agreement between the change in the dielectric constant of the composites and its fractal dimension change (characteristics of more uniform structure). The fractal dimension increases with MWCNT content up to 0.07 vol.% (Figure 9), approaching *D* = 2, which corresponds to the formation of an almost 2D structure due to BaTiO_3_ particles connected by nanotubes. At higher MWCNT contents, the fractal dimension drops together with the dielectric constant. Above the percolation threshold, the uniformity of the BaTiO_3_ distribution continues to decrease due to the attachment of BaTiO_3_ particles to carbon nanotubes and the formation of linear rows of particles; see the next section. At the same time, the apparent dielectric constant increases due to the formation of a conducting network of carbon nanotubes.

### 3.4. Mechanism of the Control over the Target Properties

**Figure 10:**
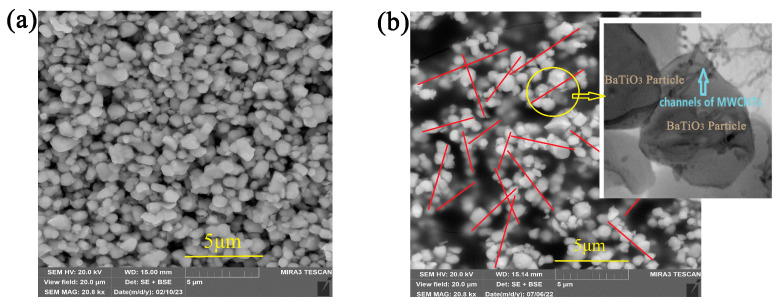
SEM images of the pre-percolation composite containing 0.07 vol.% MWCNT (**a**) and the percolating composite with 0.27 vol.% MWCNT (**b**) featuring the formation of linear structures of MWCNT-modified BaTiO_3_ particles.

The observed high sensitivity of composite fractal parameters and permittivity to the MWCNT content can be accounted for the formation of interpenetrating linear structures of BaTiO_3_ particles connected with nanotubes, as confirmed by SEM images (Figure 10). The image in Figure 10a, corresponding to the composite containing 0.07 vol.% MWCNT, features a uniform packing of the filler particles without linear arrangement and the maximum permittivity increase in the pre-percolation region. On the contrary, the image of the composite containing 0.27 vol.% MWCNT in Figure 10b indicates the formation of prominent linear structures corresponding to the interpenetrating network and percolation.

**Figure 11:**
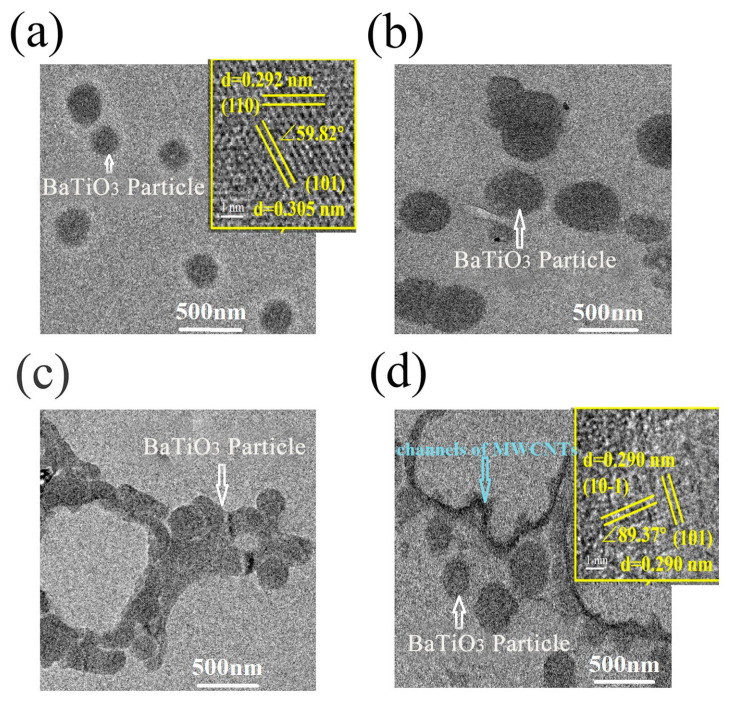
TEM images of the composites with MWCNT contents of 0.07 (**a**), 0.12 (**b**), 0.27 (**c**), and 0.36 vol.% (**d**).

TEM data (Figure 11) further confirm the considered evolution of the composite microstructure with the increase in MWCNT content. At 0.07 vol.% of MWCNTs, BaTiO_3_ particles are separated from each other by the polymer, while at 0.12 vol.%, aggregation begins, resulting in the formation of a network structure at 0.27 vol.% and their conjugation with nanotubes at 0.36 vol.%.

**Figure 12:**
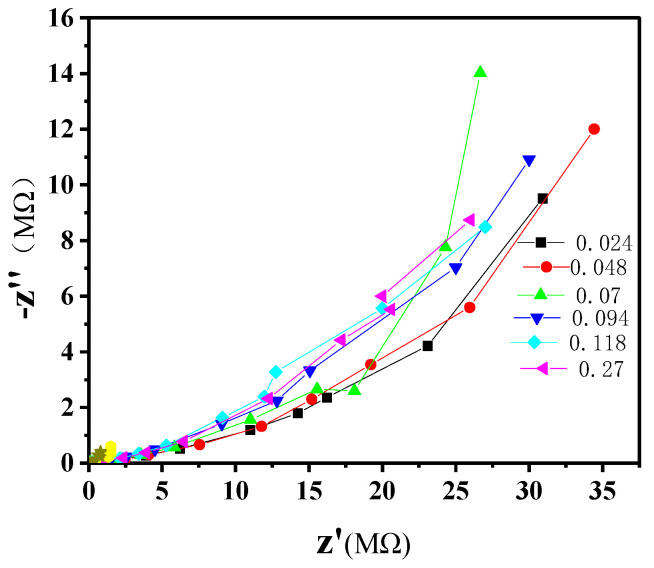
Impedance characteristics of composites with different MWCNT contents.

The impedance characteristics of the composites shown in Figure 12 as *Z*′–*Z*″ plots indicate that the sample with 0.07 vol.% MWCNT content has the most prominent growth of *Z*″ with *Z*′, reflecting the highest capacitor contribution to the impedance. This result confirms the most uniform structure of this composite with an effective separation of the filler particles and nanotubes by the polymer layers, providing the highest capacity and permittivity, as discussed above.

**Figure 13:**
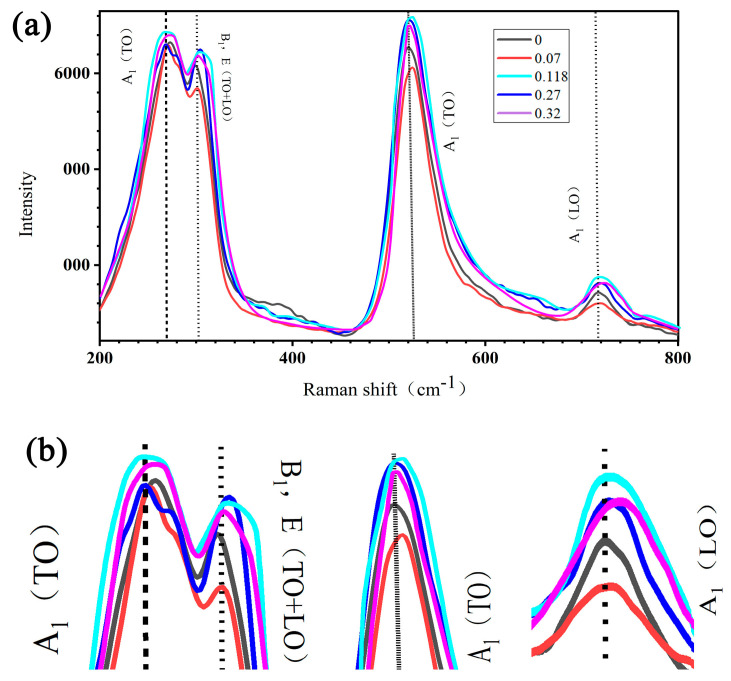
Raman spectra for composites with different MWCNT contents (0–0.32 vol.%). (**a**) Trends in peak intensity changes; (**b**) enlarged characteristic peaks of barium titanate.

The Raman spectra of the studied composites are shown in Figure 13. The main peaks corresponding to the tetragonal BaTiO_3_ phase appear at approximately 250–260, 520, and 720 cm^−1^, in agreement with [47,48]. No peaks related to MWCNTs are observed due to their low content. As shown in Figure 13b, the addition of 0.07 vol.% of MWCNTs results in permittivity increase due to structural ordering which promotes a decrease in the frequencies (red shift) and intensities of these peaks, confirming a more relaxed (less constrained and stressed) state of isolated BaTiO_3_ particles.

The increase in MWCNT content to 0.27 vol.% and above leads to the opposite trend (blue shift), indicating a more stressed state of BaTiO_3_ in tightly packed lines of particles attached to carbon nanotubes (Figure 10 and Figure 11).

## 4. Conclusions

The obtained results demonstrate a technique to significantly increase the dielectric permittivity of polymer composites without percolation due to ferroelectric filler modification with microquantities of multiwall carbon nanotubes. Depending on the quantity of MWCNT additive modifying the BaTiO_3_ filler surface, two ranges of dielectric permittivity increase in the CEPVA/(BaTiO_3_ + MWCNT) composites are observed. In the first range, permittivity grows without any considerable increase in electrical conductivity and dielectric loss at MWCNT contents up to 0.07 vol.% due to the intensification of the filler–binder interfacial interactions involving Brønsted acidic centers on the modified BaTiO_3_ surface. The growth of dielectric permittivity corresponds to the increase in fractal dimensions, confirming the formation of composite with a uniform distribution of filler particles.

The second range of dielectric constant increase at higher MWCNT concentrations is accompanied by a significant increase in electrical conductivity and is determined by the percolation in the composite with the threshold at 0.3 vol.% of MWCNTs due to the formation of conductivity pathways. Percolation is accompanied by a decrease in fractal dimensions due to the attachment of BaTiO_3_ particles to carbon nanotubes and the formation of linear rows of particles.

Therefore, the developed approach is promising for the control of the structure and properties of composites.

## Data Availability

The raw data supporting the conclusions of this article will be made available by the authors on request.
